# Supplementary material for: ResMiCo: Increasing the quality of metagenome-assembled genomes with deep learning
Source: PLoS Comput Biol. 2023 May 1;19(5):e1011001. doi: 10.1371/journal.pcbi.1011001 (PMC10174551; doi:10.1371/journal.pcbi.1011001)
Supplement: S1 Text — Fig A. Breakpoint locations for misassemblies identified by MetaQUAST (treated as ground-truth in this work). Fig B. Feature maps for four n9k-novel misassembled contigs. Fig C. Feature maps from the last layer before global pooling for four n9k-novel correctly assembled contigs. Fig D. The contig length distribution and ResMiCo performance on contigs of different lengths. Fig E. AUPRC that ResMiCo achieves on 240 subsets (differing simulation parameters) of the n9k-novel. Fig F. Distribution of errors found by MetaQUAST in the n9k-novel test dataset. Fig G. ResMiCo scores for misassembled contigs grouped by assembly error type. Fig H. ResMiCo performance measured by AUPRC on contigs from the datasets with various simulation parameters (n9k-novel). Fig I. ResMiCo performance measured by AUROC on contigs from the datasets with various simulation parameters (n9k-novel). Fig J. Number of misassemblies found by ResMiCo divided by the true number of misassemblies in the datasets with various simulation parameters (n9k-novel). Fig K. Genome fraction measured on (A) CAMI gut and (B) CAMI marine datasets before and after filtering misassembled according to ResMiCo contigs. Fig L. Features ranked by their importance. Table A. The full list of positional features computed by ResMiCo pipeline. Table B. Hyperparameters tested for ResMiCo architecture. Table C. Effect of down sampling reads on the ResMiCo predictions. Table D. Contig length cut-off effect on the ResMiCo predictions. Table E. ResMiCo performance on test data varying by the mean of the insert size distribution. Table F. ResMiCo performance on the test sets with variable stdev of the insert size distribution. Table G. The insert size distribution statistics across synthetic and real-world datasets used in this work. Table H. ResMiCo performance on two mock real-world datasets. Text A: ResMiCo’s embeddings highlight breakpoint locations. Text B: ResMiCo performance by assembly error type. Text C: NN achitecture sele [file pcbi.1011001.s001.pdf]

Supplementary Material
ResMiCo: increasing the quality of metagenome-assembled genomes with deep learning

Olga Mineeva[1,2,3,¶], Daniel Danciu[1,¶], Bernhard Schölkopf[1,2,5], Ruth E. Ley[4], Gunnar Rätsch[1,3,5,6,7,*], Nicholas D. Youngblut[4,*]

1 Department of Computer Science, ETH Zürich, Zürich, Switzerland
2 Department of Empirical Inference, Max Planck Institute for Intelligent Systems, Tübingen, Germany
3 Swiss Institute for Bioinformatics, Lausanne, Switzerland
4 Department of Microbiome Science, Max Planck Institute for Biology, Tübingen, Germany
5 ETH AI center, ETH Zürich, Zürich, Switzerland
6 Department of Biology, ETH Zürich, Zürich, Switzerland
7 Medical Informatics Unit, Zürich University Hospital, Zürich, Switzerland.

¶The authors wish it to be known that, in their opinion, the first two authors should be regarded as Joint First Authors.
* nyoungblut@tuebingen.mpg.de, gunnar.raetsch@inf.ethz.ch

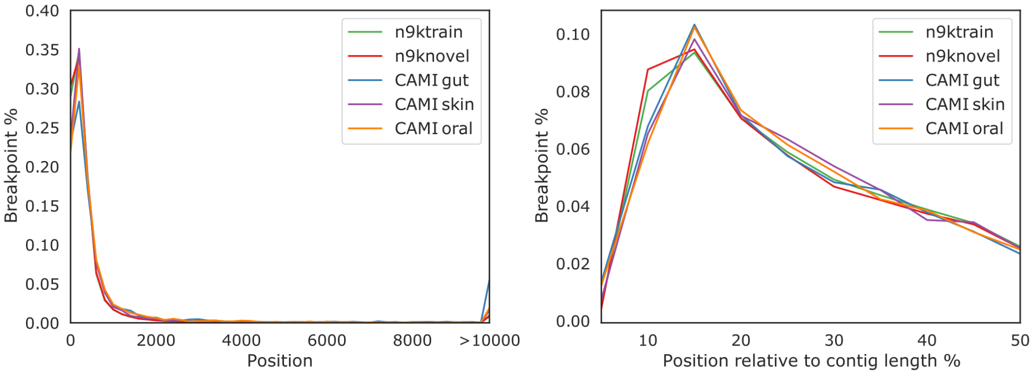

Fig A. Breakpoint locations for misassemblies identified by MetaQUAST (treated as ground-truth in this work). Left: histogram of the absolute breakpoint location for the five datasets used for training and evaluating ResMiCo. **Right:** histogram of the breakpoint location relative to the contig length for the same datasets. Breakpoint locations are computed relative to the nearest end of the contig. Approximately 9% of all misassemblies had more than one breakpoint.

Text A: ResMiCo's embeddings highlight breakpoint locations

ResMiCo maintains positional information through the convolutional layers and up to the global average pooling layer, at which point the positional information is collapsed. To assess how ResMiCo's convolved features are used for classification, we visualized the output of the last residual block (Fig **??**) by averaging the value of the 128 filters for each position. Formally, if we denote with $Z(\boldsymbol{x}) \in R^{128 \times \lfloor |x|/8 \rfloor}$ the output of the last residual block for contig $\boldsymbol{x}$, where $z_{ij}(\boldsymbol{x})$ represents the value of the ith filter at position j, we compute:

$$f_{\boldsymbol{x}}(j) = \frac{\sum_{i=1}^{128} z_{ij}(\boldsymbol{x})}{128}, j = [1 .. \lfloor |\boldsymbol{x}|/8 \rfloor]$$

where $|\boldsymbol{x}|$ represents the length of $\boldsymbol{x}$, which is shortened by a factor of 8 due to the strided convolution at the start of the last 3 residual groups (Fig **??**).

In Fig B, we plot the resulting feature maps $f_{\boldsymbol{x}}$ for several misassembled contigs $\boldsymbol{x}$ from the *n9k-novel* dataset. In each example, the breakpoint location is associated with a spike in $f_{\boldsymbol{x}}$, with the convolutional filters spreading the signal across ≈20 embedded positions around the breakpoint, suggesting that ResMiCo can be adapted to detect misassembly locations. For misassemblies with a low ResMiCo score (false negatives), the spike is shallow (Fig B, bottom-left) or (rarely) missing (Fig B, bottom-right). However, we note that spikes in the feature maps are not exclusively associated with misassembled contigs. Fig C shows several correctly assembled contigs that feature such spikes, when ResMiCo wrongly classifies them as positives.

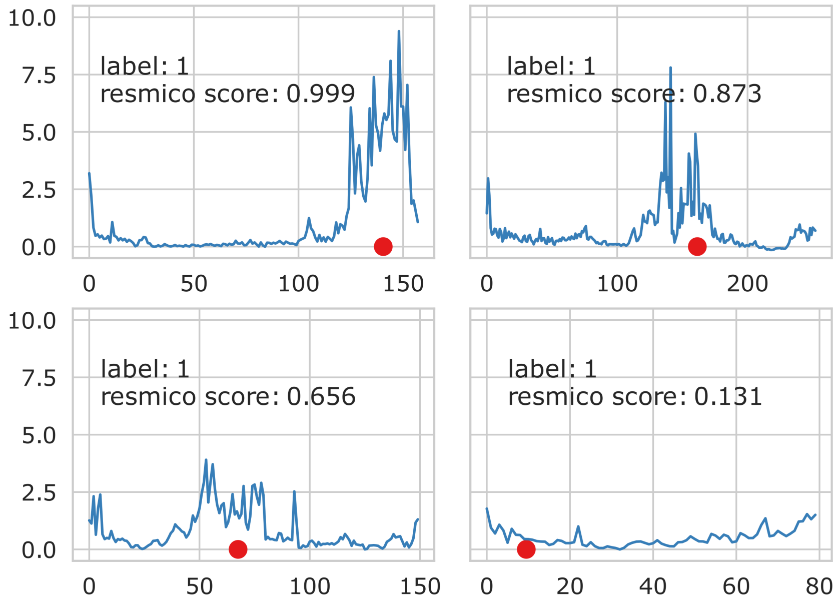

Fig B. Feature maps for four *n9k-novel* misassembled contigs. The red dot marks the breakpoint location as detected by MetaQUAST. The top row shows correctly classified contigs, and the bottom row shows false negatives.

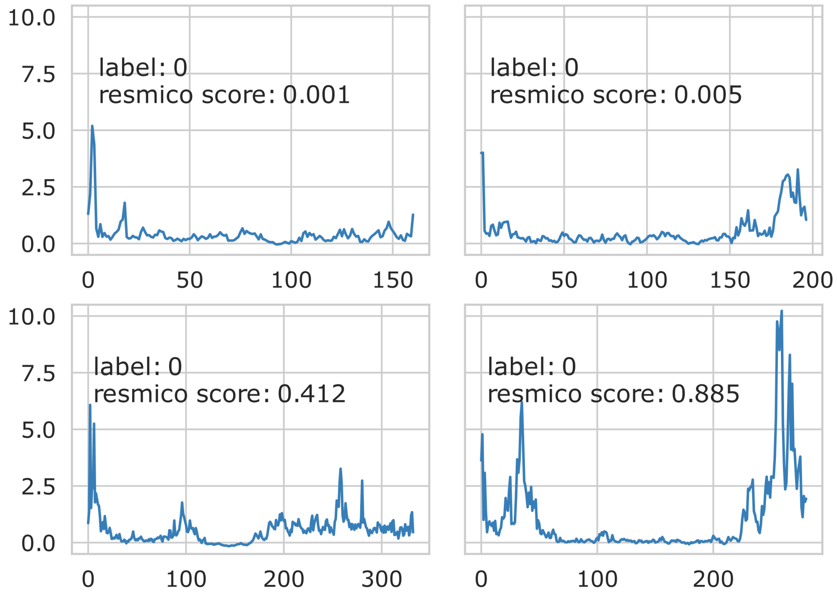

Fig C. Feature maps from the last layer before global pooling for four *n9k-novel* correctly assembled contigs. The top row shows correctly classified contigs, and the bottom row shows contigs with spikes and a false positive case (right).

ResMiCo performance relative to contigs' length

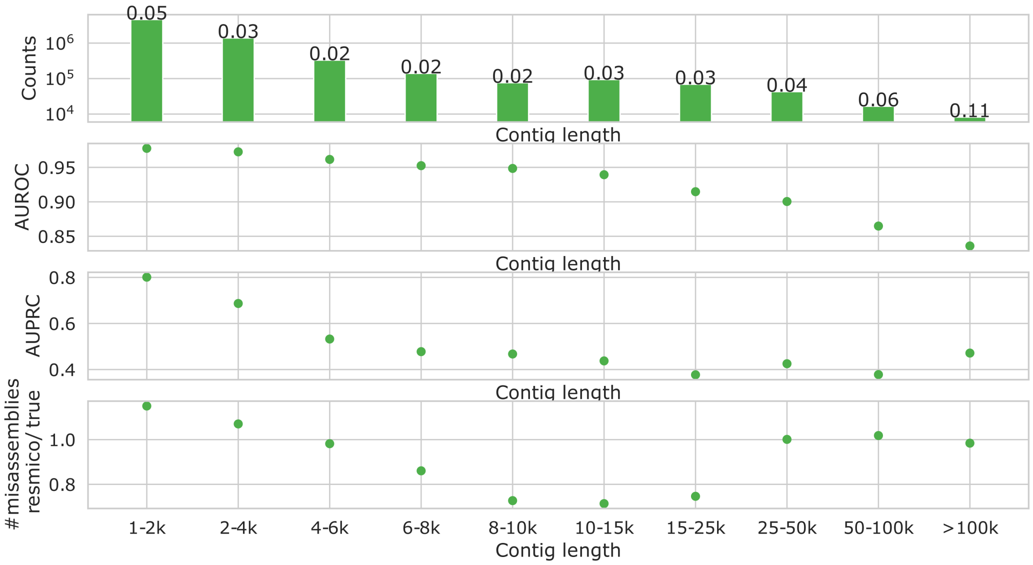

Fig D. The contig length distribution and ResMiCo performance on contigs of different lengths. The contigs were grouped according to their length. On the top plot, we showed the number of contigs within a group and indicated the proportion of misassemblies with a number on top of each bar. Next, We measured AUROC and AUPRC for each group. Long contigs are more challenging than short ones. For the threshold of 0.8, we compared the number of detected misassemblies with the true number. The bottom plot shows that the ratio is close to one.

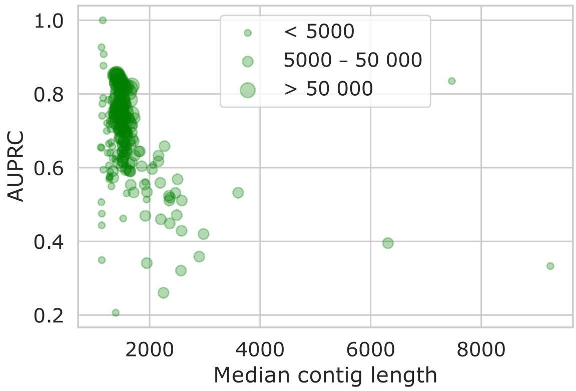

Fig E. AUPRC that ResMiCo achieves on 240 subsets (differing simulation parameters) of the *n9k-novel* test dataset plotted against the median length of contigs. Size of the marker indicates a number of contigs in the subset.

Text B: ResMiCo performance by assembly error type

MetaQUAST reports four misassembly types: interspecies translocation (`inter_transloc`), relocation (`reloc`), `inversion`, translocation (`transloc`). The distribution of misassemblies by type is shown in Fig F. Two or more misassembly types are rarely observed within one contig, so in the following analyse we used contigs containing only one type of misassembly. We plot ResMiCo scores for each error type (Fig G). As all contigs are misassembled, we would like to set all ResMiCo scores to approximately 1. Most of the misassemblies produced were interspecies translocations, which is the easiest type for ResMiCo to predict. Translocation misassemblies, happening across multiple contigs from the same genome, are the most challenging for ResMiCo.

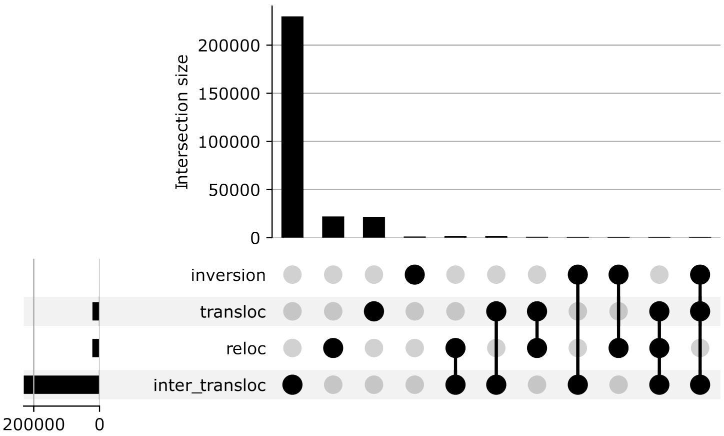

Fig F. Distribution of errors found by MetaQUAST in the *n9k-novel* test dataset.

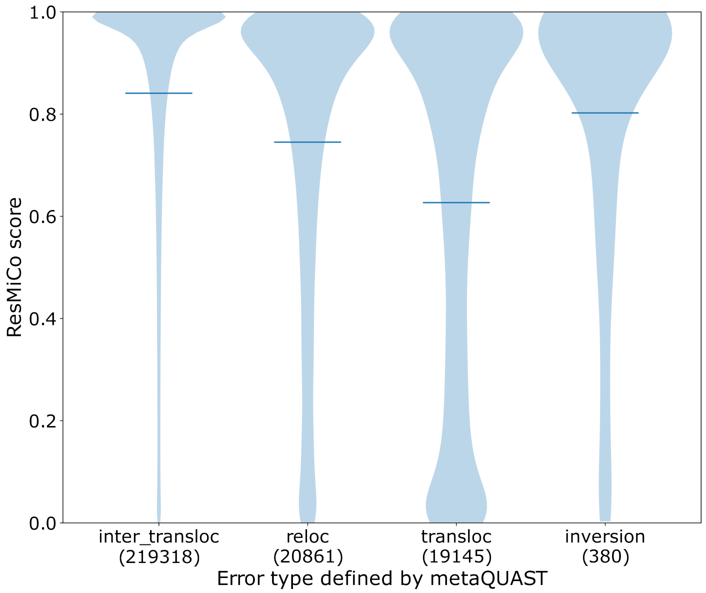

Fig G. ResMiCo scores for misassembled contigs grouped by assembly error type. Contigs are taken from the *n9k-novel* test dataset. The number of contigs in the group is indicated in parentheses. The horizontal bar shows mean score.

ResMiCo performance relative to various simulation parameters

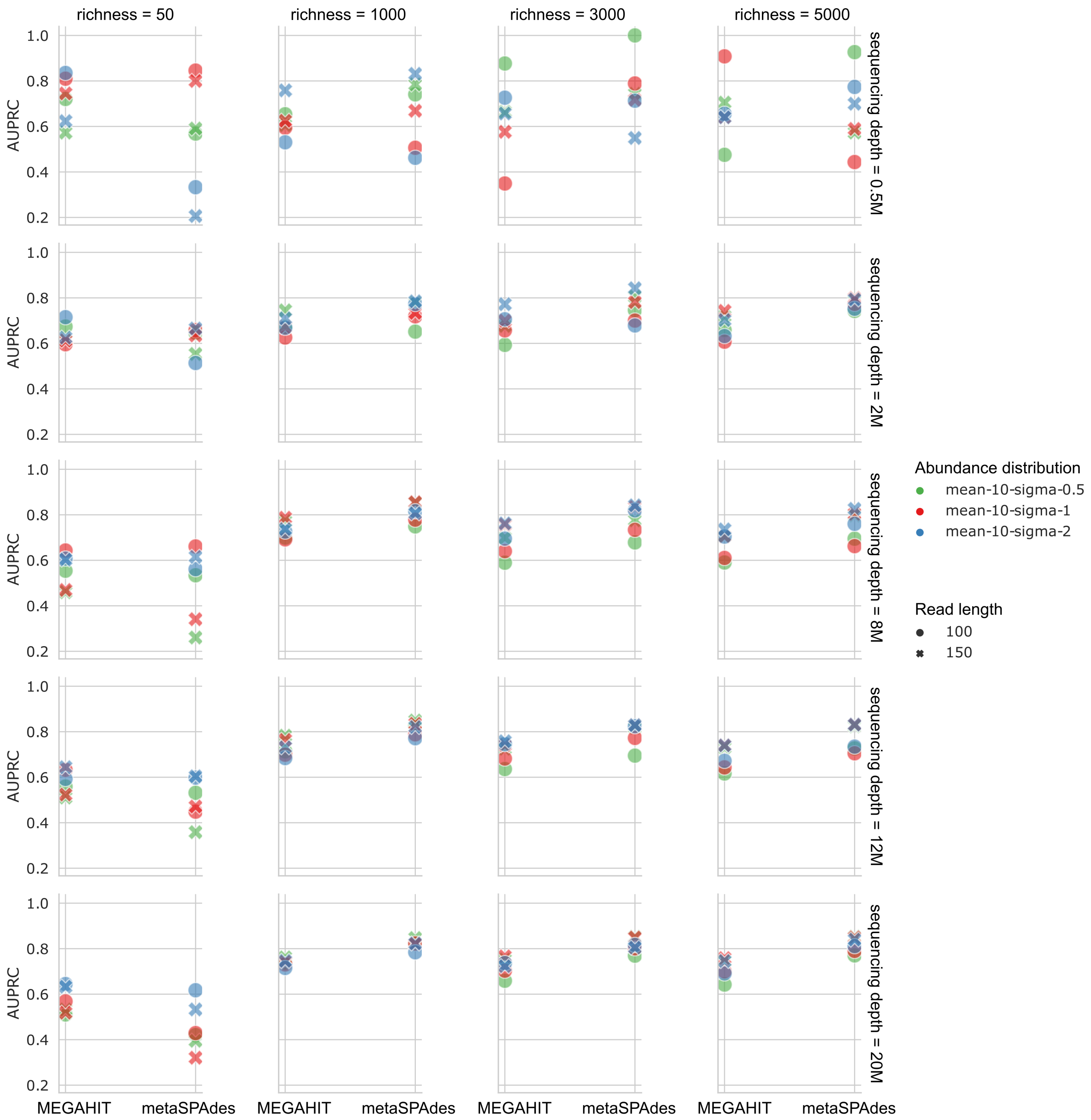

Fig H. ResMiCo performance measured by AUPRC on contigs from the datasets with various simulation parameters (*n9k-novel*). Simulation parameters include: community richness, genome abundance, read length, sequencing depth, and assembler. Low community richness and low sequencing depth are the most challenging conditions for ResMiCo, and the AUPRC varies substantially. For other parameter combinations, ResMiCo performance is stable, and AUPRC is between 0.6 and 0.8.

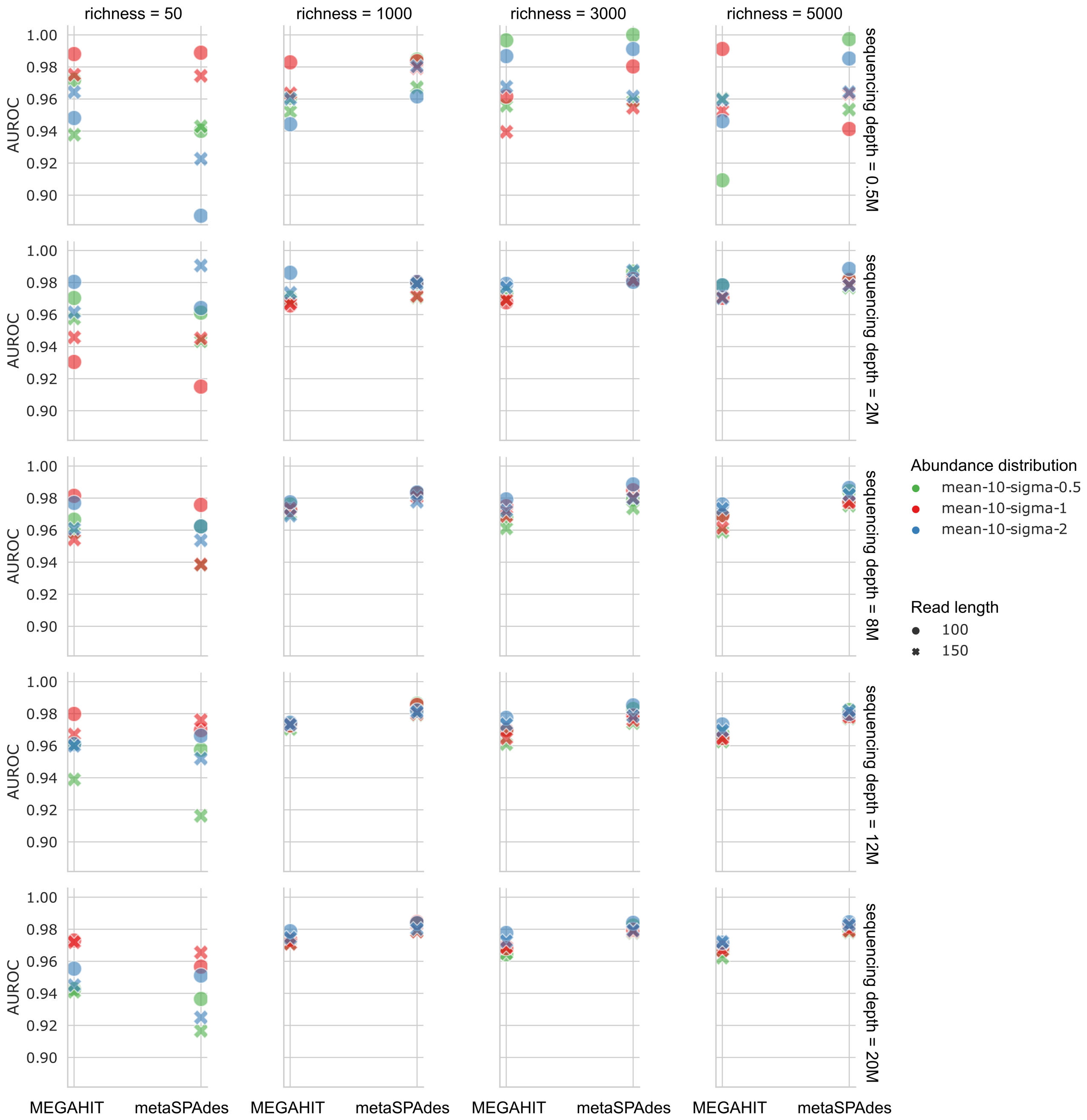

Fig I. ResMiCo performance measured by AUROC on contigs from the datasets with various simulation parameters (*n9k-novel*). Simulation parameters include: community richness, genome abundance, read length, sequencing depth, and assembler. Low community richness and low sequencing depth are the most challenging conditions for ResMiCo. For other parameter combinations, ResMiCo performance is stable, and AUROC is around 0.97.

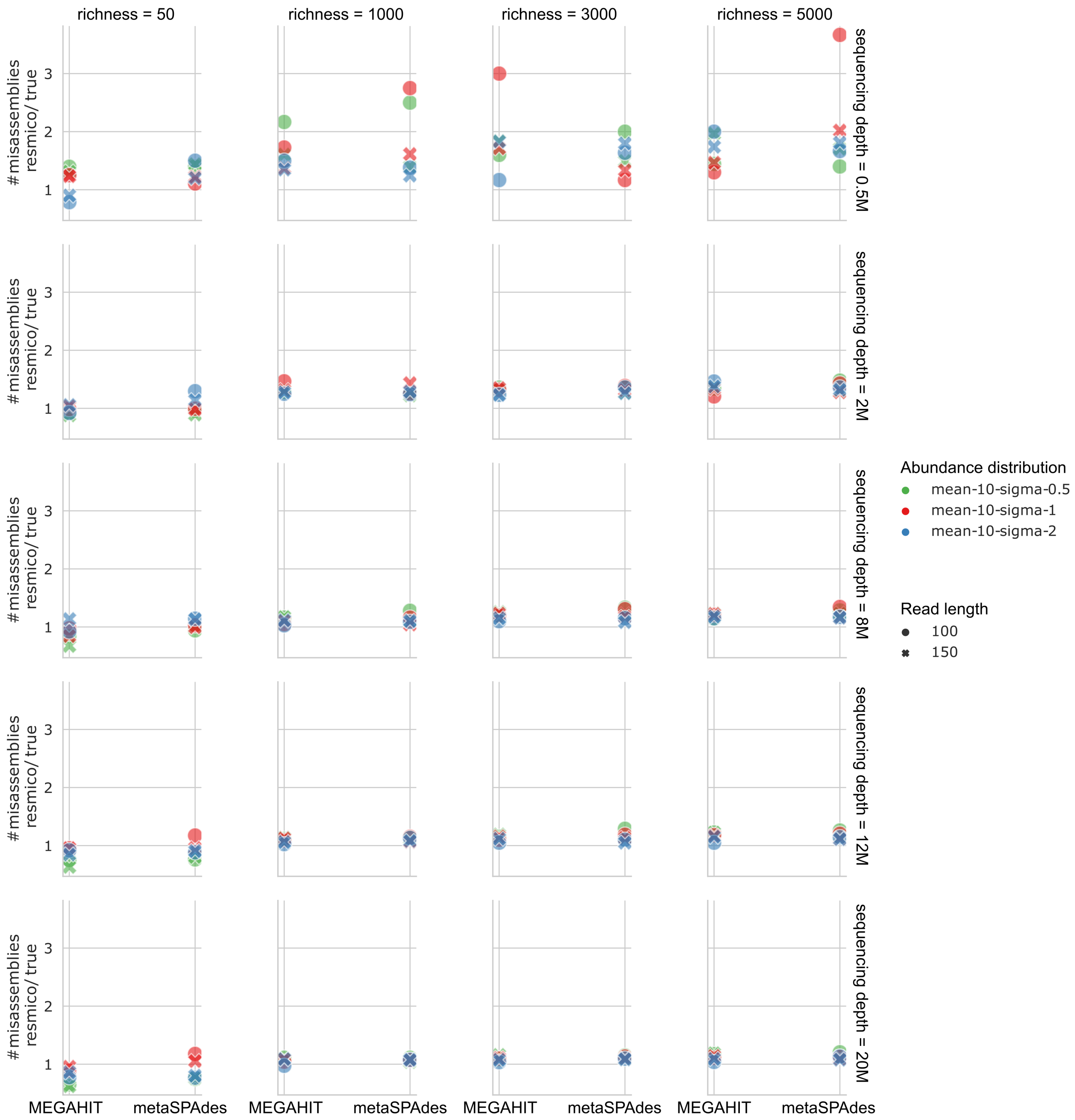

Fig J. Number of misassemblies found by ResMiCo divided by the true number of misassemblies in the datasets with various simulation parameters (*n9k-novel*). Simulation parameters include: community richness, genome abundance, read length, sequencing depth, and assembler. ResMiCo tends to overestimate error rate when sequencing depth is low (0.5M). For other parameter combinations, ResMiCo estimate is close to the observed error rate. Threshold of 0.8 was used here.

Genome fraction

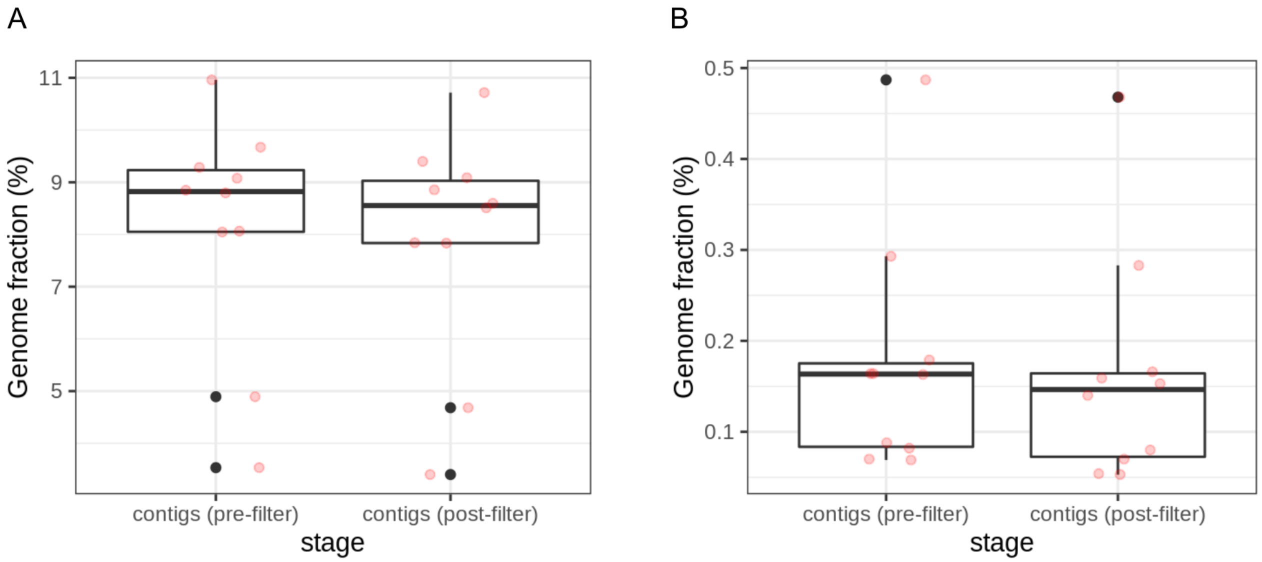

Fig K. Genome fraction measured on (A) CAMI gut and (B) CAMI marine datasets before and after filtering misassembled according to ResMiCo contigs. The genome fraction did not substantially change for either dataset when comparing post- versus pre-contig filtering by ResMiCo (Wilcox, P $\geq$ 0.66 for both datasets). Red points denote values for individual metagenomes.

Feature selection by their SHAP importance

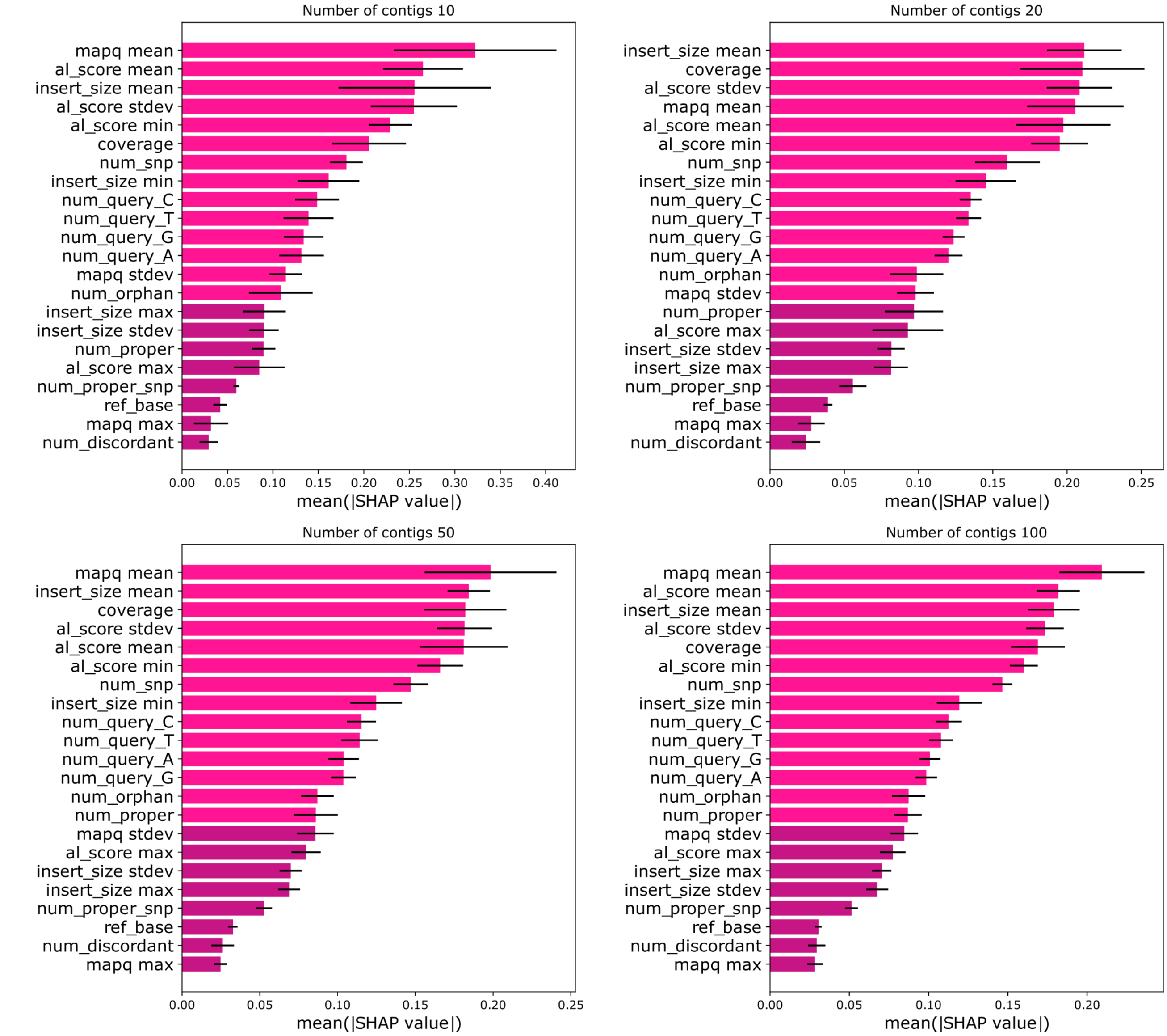

Fig L. Features ranked by their importance. We ran the SHAP method for 10, 20, 50, 100, and 200 contigs (Fig 3); for simplicity, the same number of contigs were given to SHAP as a background and to generate SHAP explanations. While absolute values of importance vary, the overall ranking remain stable. We note that the less important features were exactly the same for the runs with 50, 100, and 200 contig, which shows that 200 contigs are sufficient to determine which features yo discard. The runs with 10 and 20 contigs disagree only in one feature. The lighter color denotes features used by the ResMiCo model. `mapq` and `al_score` are mapping quality and alignment score, as defined by Bowtie2. `num_snp` is the number of SNVs among aligned reads relative to the reference. `num_query_[ATGC]` is the base composition of aligned reads at the target position. `num_orphan` is the number of aligned reads in which only one of the pairs aligns properly. `num_proper` is the number of read pairs that align properly, as defined by Bowtie2. `num_proper_snp` is properly aligned reads with a SNV relative to the reference at the target position. `ref_base` is the reference base `[ATGC]` at the target position. Error bars correspond to the stdev computed over 5 runs.

Features generated for ResMiCo training

Feature	Description	Aggregation	Match	Preproc.	Used
coverage	Number of reads aligned to position	Count	A	Std	Y
ref_base	Nucleotide in the reference genome	-	-	Onh	N
num_query	Number of A/C/G/T nucleotides mapped to position	Count	A	Nrm	Y
num_snp	Number of aligned bases different than ref_base	Count	A	Nrm	Y
num_proper	Number of pair-matched aligned reads	Count	M	Nrm	Y
num_proper_snp	Number of SNPs from pair-matched aligned reads	Count	A	Nrm	N
num_discordant	Number of un-matched aligned reads	Count	A	Nrm	N
num_orphan	Number of orphan aligned reads	Count	M	Nrm	Y
al_score	Bowtie alignment scores	**Min**, **Mean**, Max, **Stdev**	M	Std	Y
insert_size	Length of the aligned inserts	**Min**, **Mean**, Max, Stdev	M	Std	Y
mapq	Bowties mapping quality of the aligned reads	Min, **Mean**, Max, Stdev	M	Std	Y

Table A. The full list of positional features computed by ResMiCo pipeline. For features computed by aggregating over multiple reads aligned to a position in a contig, the third column lists the type of aggregation, and the fourth column indicates if the feature was computed only across reads that matched the reference base (M) or for all reads (A). The fifth column states preprocessing applied to the feature: standardization (Std), normalization (Nrm), and one-hot encoding (Onh). The last column indicates if the feature was used for training the network presented in this paper: yes (Y), no (N). The corresponding type of aggregation used is in bold.

Text C: NN achitecture selection

The hyperparameter search for selecting the best-performing ResMiCo architecture was done by evaluating each model's performance on the validation set (i.e., randomly sampled 10% of the training set).

First, we selected the best performing neural network architecture type. For this, we constructed a deep convolutional NN, a bidirectional recurrent NN with LSTM units and with GRU units, a transformer encoder, and a residual convolutional NN. All models had roughly 0.5 million trainable weights and were trained on a subset of the *n9k-train* dataset (20%). The residual convolutional neural network outperformed the other network architectures for all contig lengths and was thus selected for this work. All subsequent hyperparameter optimizations were applied to this architecture only. Due to resource limitations, we did not test all combinations of hyperparameters but rather selected the most promising ones iteratively. The number of RBs and RGs were determined first, then other hyper-parameters combinations were tested.

**Parameter**	**Values**
Number of residual blocks (RB) in Residual groups (RG)	{[2,5,2], **[2, 5, 5, 2]**, [2, 3, 5, 5, 2], [2, 3, 5, 5, 3, 2]}
Number of filters for the first convolution (Conv) layer	$\{4, \mathbf{16}\}$
Kernel size for Conv layers in RB	$\{3, \mathbf{5}\}$
Aggregation along the spatial axis	{**global average pooling**, global max pooling, concatenated output of max and average global pooling }
Number of hidden units in fully connected (FC) layers n_h	$\{\mathbf{50}, 100\}$
Number of FC layers n_h	$\{\mathbf{1}, 2\}$
Initial learning rate	$\{\mathbf{0.0001}, 0.001\}$
Maximum input length	$\{5000, 10000, \mathbf{20000}\}$

Table B. Hyperparameters tested for ResMiCo architecture. The final choices for ResMiCo architecture are marked bold.

Text D: Effect of the length cut-off and the read down-sampling on ResMiCo predictions

We studied how the down-sampling of reads and different contig length cut-offs influence contig representation and ResMiCo prediction scores. We simulated data as done for the *n9k-novel* dataset, with the following reduced set of parameters: i) richness of 1000 or 5000 genomes, ii) lognormal abundance distribution with a mean of 10 and a sigma of 1 or 2, iii) read lengths of 150bp, and iv) a sequencing depth of 2 or 8 million paired-end reads. There were a total of 96 parameter combinations. In the first experiment, we down-sampled reads to five levels (0.5, 0.1, 0.05, 0.01, 0.005) and generated features for each level. ResMiCo was then applied to the contigs shared across all levels ($n = 2655$). Table C shows that the AUPRC and the ratio between the predicted error rate and the true error rate is stable across levels. Only 0.8% of the contigs changed prediction class across levels. In the second experiment, contigs shorter than a specified cut-off were discarded before read mapping. Contigs longer than the largest cut-off (10k) shared between all simulations were used to test ResMiCo. While 1% of contigs were assigned the opposite classes in different cut-off datasets, ResMiCo performance remained stable overall (Table D). Taken together, these results indicate that ResMiCo performance is robust to contig subsampling and filtering by contig length.

% contigs kept	100	50	10	5	1	0.5
AUPRC	0.79	0.79	0.79	0.79	0.79	0.78
# misassemblies predicted / true	1.21	1.23	1.23	1.25	1.24	1.24

Table C. Reads down-sampling effect on the ResMiCo predictions

Length cut-off	1000	1500	2000	3000	5000	10000
AUPRC	0.36	0.38	0.37	0.36	0.37	0.36
# misassemblies predicted / true	0.94	0.95	0.93	0.89	0.86	0.88

Table D. Contigs length cut-off effect on the ResMiCo predictions

Text E: ResMiCo sensitivity to changes in insert size distribution

We evaluated the robustness of ResMiCo to varying insert size distributions, given that paired-end read insert size can substantially affect assembly quality and read mapping statistics. For this experiment, we utilized the same parameter subset as done for our contig subsampling and length cutoff evaluation (see above), except we changed the insert size distributions to those shown in Tables E and F. We note that the *n9k-train* dataset consisted of 4 insert size distribution parameter sets: mean=190 & stdev=75, mean=270 & stdev=50, mean=350 & stdev=75, and mean=450 & stdev=120.

The AUCPR and predicted misassembly rate were quite similar to performance on the *n9k-novel* dataset, with the insert size mean varying from 180 to 380 (Table E). For the mean of 600, we observed a drop of the AUCPR to 0.48, and the misassembly rate was overestimated by 2.45x. With a fixed mean of 270 and varying stdev, ResMiCo performance starts to degrade when the stdev increased to 250 (Table F). When used large mean and stdev values (500 and 130), AUPRC was 0.59. Altogether, these results suggest that ResMiCo is sensitive to mean insert size distribution ranges outside of the training dataset. For this reason, we designed inclusion criteria for evaluating real metagenomes with ResMiCo based on the insert size distribution statistics of the target metagenomes (see Data preprocessing).

Mean, Stdev of insert size distribution	180, 50	200, 50	230, 50	270, 50	320, 50	380, 50	600, 50
AUPRC	0.69	0.69	0.71	0.71	0.74	0.72	0.48
# misassemblies predicted / true	1.05	1.08	1.12	1.13	1.17	1.12	2.45

Table E. ResMiCo performance on test data varying by the *mean* of the insert size distribution. ResMiCo performance declines at the extreme values of the mean insert size distribution gradient.

Mean, Stdev of insert size distribution	270, 30	270, 50	270, 70	270, 90	270, 110	270, 250
AUPRC	0.75	0.71	0.71	0.72	0.68	0.63
# misassemblies predicted / true	1.16	1.13	1.22	1.23	1.23	1.62

Table F. ResMiCo performance on the test sets with variable *stdev* of the insert size distribution. ResMiCo performance slightly decreases with increasing stdev, but AUPRC remains high (0.68) up to a stdev of 110.

Dataset	mean	stdev	q5	q95
n9k-train	284	94	167	462
n9k-novel	258	44	175	305
Cami gut	260	35	202	281
Cami skin	261	34	162	282
Cami oral	254	45	161	281
Cami marine	253	70	146	283
Cami plant	258	56	182	281
UHGG	226	80	134	341
TwinsUK	294	67	130	356
Animal-gut	246	67	114	335
MarineMetagenomeDB	257	104	118	413
Mantri2021	320	101	103	427
Pinnell2019	249	72	139	352
TerrestrialMetagenomeDB	233	88	0	357

Table G. The insert size distribution statistics across synthetic and real-world datasets used in this work. The table shows values computed over the whole dataset. On the metagenome level for real-world datasets, we observed extreme cases of q5=0 & q95=600. We didn't apply ResMiCo on such samples, because they didn't meet inclusion criteria (see Data preprocessing).

ResMiCo evaluation on real-world mock communities

Dataset	Project accession	Contigs	Total length	Misassemblies	TP	FP	AUROC	AUPRC
BMock12	SRR8073716	3907	28.2 Mbp	30	23	38	0.987	0.718
MBARC-26	SRR3656745	9626	46.7 Mbp	92	80	2115	0.915	0.233

Table H. ResMiCo performance on two mock real-world datasets. ResMiCo score cutoff was 0.8. *TP* is the number of misassembled contigs correctly identified by ResMiCo, while *FP* is the number of correctly assembled contigs wrongly predicted to be misassembled by ResMiCo.

Text F: Clustering of misassemblies

We clustered contigs based on their representations in the ResMiCo model and assessed whether these clusters matched the grouping of contigs by misassembly type. A strong overlap of these 2 clustering methods would suggest that the misassembly type could be classified simply by using the clustering of model representations.

We note that there are 4 types of misassemblies reported by MetaQUAST: interspecies translocation, relocation, inversion, translocation. Inversions were only 0.1% of all misassemblies, so we did not include them in the following analysis.

To represent contigs as vectors of the same size, we produced embeddings from the ResMiCo pooling layer (the same as for UMAP). We randomly sampled 1 million contigs from each dataset and used only misassemblies for the following analysis. We used k-means clustering to generate 3 clusters for the *n9k-train* dataset and predicted cluster assignments for the n9k-novel test dataset. To compare the two clusterings (based on misassembly type and on k-means from the embeddings), we computed Clustering Purity (0.52) and Adjusted Rand Index (0). These finds demonstrate that the model embeddings do not strongly correspond with misassembly types and so one can not use clustering of the embeddings to determine misassembly type for real-world data.

To get some insights into contig characteristics that are the most challenging for ResMiCo, we also fitted k-means with 50 clusters. Clusters with less than 100 contigs were filtered out for clarity of the results, so the resulting number of clusters was 35 clusters. We describe contigs with features used to simulate the data (i.e., sequencing depth, community richness, assembler type, and read length), with misassembly types reported by MetaQUAST (i.e., interspecies translocation, relocation, and translocation) and the most informative (according to SHAP) positional features (i.e., mapping quality, mean alignment score, mean insert size, aand coverage), and also contig length. The Table S2 shows mean values of each characteristic within cluster and is sorted based on average ResMiCo score. At the bottom of the table, there are clusters of misassemblies that are challenging to accurately detect with ResMiCo. These clusters have the following characteristics: high median contig length, more translocation misassembly types, and low richness. The same can be concluded from the other figures (Fig D & E for contig length, Fig G for misassembly type, and Fig I for richness) and is discussed in the Results section. However, there is, for instance, Cluster 29 with a an average ResMiCo score of 0.53, but from the high level characteristics, it looks similar to the clusters with high average score.

Text G: Optimizing data generation performance

ResMiCo's data simulation pipeline generates features for training the ResMiCo model by re-aligning reads against the putative contigs and extracting statistics for each positions from the realigned reads (Fig 1D). Since the original implementation of the feature generation pipeline, based on pysam+htslib, was prohibitively slow for manipulating the amount of data that ResMiCo required for training, we wrote our own pileup generation and feature extraction library. ResMiCo's feature generation is based on the BamTools (`https://github.com/pezmaster31/bamtools`) library that provides raw access to the BAM files generated by Bowtie2 [1]. The speed gain was achieved by loading all alignments in memory into re-sizable chunks (based on the machine's available RAM) rather than streaming data from disk. Also, rather than first generating a pileup and then analyzing the resulting pileup file, the two steps were unified (i.e., the features were computed on the fly at the time of the pileup generation). The contigs in BAM files were read and processed in parallel (since they are independent of each other), thus making effective use of all CPU cores. Writing data to disk was delegated to a separate consumer thread, thus avoiding slow IO blocking pileup generation. In addition, the resulting features were written on disk in a binary format with a fixed schema, which allowed both efficient storage and indexing. The modified feature generation was about an order of magnitude faster than the original pysam+htslib-based feature generation. The source code for the feature generation library is available at `https://github.com/leylabmpi/ResMiCo/tree/master/ResMiCo-SM/feature_extractor`.

References

[1] Langmead B, Salzberg SL. Fast gapped-read alignment with Bowtie 2. Nat Methods. 2012;9(4):357–359.
