# Supplementary material for: ResMiCo: Increasing the quality of metagenome-assembled genomes with deep learning
Source: PLoS Comput Biol. 2023 May 1;19(5):e1011001. doi: 10.1371/journal.pcbi.1011001 (PMC10174551; doi:10.1371/journal.pcbi.1011001)
Supplement: Attachment — Submitted filename: renamed_4c385.pdf [file pcbi.1011001.s004.pdf]

Reviewer Response for “ResMiCo: increasing the quality of metagenome-assembled genomes with deep learning”

The authors describe ResMiCo, a novel method for reference- free detection of misassemblies using the residual convolutional neural network. Authors have generated a large and complex training dataset which accounts for multiple variables. It was shown that ResMiCo has an improved performance compared to existing methods and reasons behind these improvements were discussed. They are: 1) a significantly larger and more complex training dataset; 2) More and better selected features; and 3) use of deeper convolutional networks made possible by the introduction of residual blocks. ResMiCo is indeed a significant improvement in the field and has potential features such as indicating misassembly locations which could make it even more useful.

1) The manuscript lacks a detailed description of the type/nature of simulated misassemblies. Was the setup designed to let the assemblers do all types of misassemblies that were then detected using metaQUAST and the reference genomes? This needs to be discussed in more detail in methods and the discussion part should include a discussion of the types of misassemblies ResMiCo can detect and the limitations it has.

2) Another important question is whether ResMiCo is able to detect chimeric contigs assembled from related organisms’ sequences. It should either be stated that it doesn’t detect such misassemblies or a section of a manuscript should be added to show how it was benchmarked and what the performance was.

3) Limitations of the range of domains of life for which ResMiCo can detect misassemblies should also be discussed/mentioned.

4) The benchmarking of ResMiCo on real metagenome datasets was limited to gut samples only. Adding samples from other environments such as marine and soil that have different community complexities would make the benchmark more persuasive.

5) More analysis of the correlation of true and ResMiCo’s error rates with different assembly parameters should be done to check for systematics biases. From what is there now, the statement on N50 as an assembly quality measure in Fig 5b needs more elaboration as there is 0.9 correlation between true error rate and N50 where samples are actually well assembled (richness=50, seq_depth=8000000) which can also be seen from a completely non-overlapping y-axis for N50 (1-3k vs 5-25k). Also, is it correct to assume from Fig 5a that ResMiCo detects approximately twice the true error rate? The effect of this needs to be addressed and explained.

6) Specification of compute and memory requirements and comparison to other tools is missing in the manuscript. Especially, given the note in the GitHub page about the strong preference for GPU rather than CPU for running ResMiCo it is necessary to specify how much of compute resource it takes to run per metagenome assembly.

7) L221 “The prediction for the contig was obtained by selecting the maximum score across all chunks” - The true probability of having a misassembly in a contig is not equal to a maximum probability of one of 20 kbp chunks of a contig. Therefore, it should be a product of probabilities that there is no misassembly in the chunk (1-x). For example, if there are two chunks with 60% and 40% probability of misassembly then the combined probability should be,

$$1 - (1 - 0.6) * (1 - 0.4) = 0.76, 76\%$$

   Another issue is adjusting this to strides between chunks. In addition, it is also not explained why there should be a stride between chunks of long contigs.

   When this change is introduced the percentage of misassembled contigs will probably increase.

8) In the manuscript authors use the absolute counts of misassembled contigs for statistical purposes. However, it is possibly more accurate to normalise the misassembly rate per length (kbp) of contigs unless authors can provide reasons for why looking only at the absolute count is sufficient.

9) I could not install ResMiCo and below are the outputs of commands as described in the github repo.

   Attempt to install via conda:

```
$ conda create -n resmico_env bioconda::resmico
Collecting package metadata (current_repodata.json): done
Solving environment: failed with repodata from
current_repodata.json, will retry with next repodata
source.
Collecting package metadata (repodata.json): done
Solving environment: -
```

   Attempt 2 to install via conda:

```
$ conda create -n resmico_env resmico
Collecting package metadata (current_repodata.json): done
```

```
Solving environment: failed with repodata from
current_repodata.json, will retry with next repodata
source.
Collecting package metadata (repodata.json): done
```

Attempt to install via pip:

```
$ pip install resmico
Collecting resmico
ERROR: Could not find a version that satisfies the
requirement resmico (from versions: none)
ERROR: No matching distribution found for resmico
```

Other minor comments [with square brackets for suggested edits]:

1) Note in GitHub could be more clear: “Note: Although ResMiCo can be run on a CPU, the performance is orders of magnitude wors[e than on a GPU], so we only recommend running on CPU for testing.”
2) FigS1 - add a note that these are “true” misassemblies predicted by MetaQUAST.
3) L55 “collection [of] gut metagenomes”
4) L76 Please add the explanation that the “insert size” is the distance between two paired reads as this might not be well known to everyone and might lead to misunderstanding that it is the size of insertion that is a misassembly.
5) Mistake on L269 - “the evaluation [of] metagenomics analysis tools.”
6) L93 “The pool was also filtered by CheckM-estimated completeness (≥ 90%) and contamination (≤ 5%). Other filtering criteria included: i) only ”high” MIMAG quality” - It is redundant to mention “CheckM-estimated completeness (≥ 90%) and contamination (≤ 5%)” filters together with “high” MIMAG quality standard as these CheckM filters are included in “high” MIMAG standard.
