# Supplementary material for: ResMiCo: Increasing the quality of metagenome-assembled genomes with deep learning
Source: PLoS Comput Biol. 2023 May 1;19(5):e1011001. doi: 10.1371/journal.pcbi.1011001 (PMC10174551; doi:10.1371/journal.pcbi.1011001)
Supplement: Attachment — Submitted filename: response_to_reviewers.pdf [file pcbi.1011001.s005.pdf]

Editor comments

Dear Dr. Youngblut,

Thank you very much for submitting your manuscript "ResMiCo: increasing the quality of metagenome-assembled genomes with deep learning" for consideration at PLOS Computational Biology.

As with all papers reviewed by the journal, your manuscript was reviewed by members of the editorial board and by several independent reviewers. In light of the reviews (below this email), we would like to invite the resubmission of a significantly-revised version that takes into account the reviewers' comments.

The reviewers all agree that the methods are an improvement over the state-of-the-art and that the tool could potentially be widely used, but raise several important points related to both unclear presentation and whether some of the conclusions are sufficiently supported. Of particular interest to us are the concerns related to the evaluation on real data: the suggestion of reviewer #3 to test ResMiCo on data with a known standard seems very reasonable; similarly, the comment of reviewer #1 that only human gut samples are used should be addressed (ideally by including additional benchmarks).

We cannot make any decision about publication until we have seen the revised manuscript and your response to the reviewers' comments. Your revised manuscript is also likely to be sent to reviewers for further evaluation.

When you are ready to resubmit, please upload the following:

[1] A letter containing a detailed list of your responses to the review comments and a description of the changes you have made in the manuscript. Please note while forming your response, if your article is accepted, you may have the opportunity to make the peer review history publicly available. The record will include editor decision letters (with reviews) and your responses to reviewer comments. If eligible, we will contact you to opt in or out.

[2] Two versions of the revised manuscript: one with either highlights or tracked changes denoting where the text has been changed; the other a clean version (uploaded as the manuscript file).

Important additional instructions are given below your reviewer comments.

Please prepare and submit your revised manuscript within 60 days. If you anticipate any delay, please let us know the expected resubmission date by replying to this email. Please note that revised manuscripts received after the 60-day due date may require evaluation and peer review similar to newly submitted manuscripts.

Thank you again for your submission. We hope that our editorial process has been constructive so far, and we welcome your feedback at any time. Please don't hesitate to contact us if you have any questions or comments.

Sincerely,

Luis Pedro Coelho
Academic Editor
PLOS Computational Biology

William Noble
Section Editor
PLOS Computational Biology

****

The reviewers all agree that the methods are an improvement over the state-of-the-art and that the tool could potentially be widely used, but raise several important points related to both unclear presentation and whether some of the conclusions are sufficiently supported. Of particular interest to us are the concerns related to the evaluation on real data: the suggestion of reviewer #3 to test ResMiCo on data with a known standard seems very reasonable; similarly, the comment of reviewer #1 that only human gut samples are used should be addressed (ideally by including additional benchmarks).

Reviewer's Responses to Questions

Comments to the Authors:
Please note here if the review is uploaded as an attachment.

We thank the editor and reviewers for their very helpful comments, which have resulted in a much improved manuscript and bioinformatic tool (the ResMiCo Python package). As described below, we greatly expanded the size of the training dataset and added many new evaluations of our updated ResMiCo model. All figures and tables regarding our new model have been updated accordingly. Simulating and training a new model on a substantially larger training dataset resulted in a number of computational challenges, which required considerable time to address. Hence we required an extension to fully complete the model re-training, all subsequent evaluations of the new model, and revision of the manuscript. As we show in the updated manuscript, the new model is substantially more robust in certain aspects relative to the original model, which was already a pronounced improvement versus the state of the art.

Reviewer 1

The authors describe ResMiCo, a novel method for reference- free detection of misassemblies using the residual convolutional neural network. Authors have generated a large and complex training dataset which accounts for multiple variables. It was shown that ResMiCo has an improved performance compared to existing methods and reasons behind these improvements were discussed. They are: 1) a significantly larger and more complex training dataset; 2) More and better selected features; and 3) use of deeper convolutional networks made possible by the introduction of residual blocks. ResMiCo is indeed a significant improvement in the field and has potential features such as indicating misassembly locations which could make it even more useful.

We thank the reviewer for their encouraging comments.

1) The manuscript lacks a detailed description of the type/nature of simulated misassemblies. Was the setup designed to let the assemblers do all types of misassemblies that were then detected using metaQUAST and the reference genomes? This needs to be discussed in more detail in methods and the discussion part should include a discussion of the types of misassemblies ResMiCo can detect and the limitations it has.

We added a section on "ResMiCo performance by assembly error types" to the supplement, where we show the distribution of misassembly types and ResMiCo performance for each assembly error type. ResMiCo is able to identify most of the *interspecies translocations*, while within-genome *translocation* misassemblies are the most challenging to detect (Fig S7).

2) Another important question is whether ResMiCo is able to detect chimeric contigs assembled from related organisms' sequences. It should either be stated that it doesn't detect such misassemblies or a section of a manuscript should be added to show how it was benchmarked and what the performance was.

Misassemblies that produce chimeric contigs are a misassembly type detected by MetaQUAST, and so ResMiCo can detect this type of misassembly. We have added this clarification at the end of the "Simulated data" subsection of the Materials and Methods. In the Supplementary Material, we also show that most of the misassemblies produced via our simulation pipeline are chimeric contigs (e.g., interspecies translocations; see Fig S6). ResMiCo shows the highest accuracy for detecting this type of assembly error (Fig S7).

We also simulated a test dataset ("n2k-novel-intraspecies") with substantially more genomes per species than the n9k-novel test dataset (33.3 +/- 115 versus 2.4 +/- 13.7). ResMiCo performance on n2k-novel-intraspecies declined relative to n9k-novel (AUPRC 0.487, AUROC 0.955), which was likely due to more challenging inter-genome translocations among very closely related taxa. Still, ResMiCo outperformed the closest

competitor: metaMIC (AUPRC 0.080, AUROC 0.805). These results are now described in the Results subsection entitled "ResMiCo outperforms existing models and is robust to metagenome novelty".

3) Limitations of the range of domains of life for which ResMiCo can detect misassemblies should also be discussed/mentioned.

We have updated the Discussion (second-to-last paragraph) to clarify that while we did use a very broad selection from the bacterial and archaeal tree of life for model training/testing, future work could include other domains of life (i.e., eukaryotes and viruses). While this is a very interesting avenue of research, the added challenges of such research place it outside of the scope of this current project.

4) The benchmarking of ResMiCo on real metagenome datasets was limited to gut samples only. Adding smples from other environments such as marine and soil that have different community complexities would make the benchmark more persuasive.

We have added benchmarking of ResMiCo on 2 additional CAMI datasets that were simulated with genomes from marine (CAMI2 marine; https://frl.publisso.de/data/frl:6425521/marine/) and plant-associated (CAMI2 plant-associated; https://frl.publisso.de/data/frl:6425521/plant_associated/) biomes. The performance of ResMiCo was comparable to the previously accessed human-associated CAMI datasets (CAMI2 marine: AUPRC = 0.831, AUROC = 0.990; CAMI2 plant-associated: AUPRC = 0.611, AUROC = 0.965). We have updated the Results to include these new findings.

In addition to the newly added CAMI datasets, we have added ResMiCo benchmarking on 4 real metagenome datasets from non-human biomes: Pinnell2019, Mantri2021, MarineMetagenomeDB, and TerrestrialMetagenomeDB (see Materials and Methods). We observed that these new datasets included Illumina paired reads with insert size distributions substantially more variable than in the CAMI datasets, which have been frequently used for benchmarking bioinformatic tools (e.g., metaMIC). For instance, in the MarineMetagenomeDB dataset, there are samples with a q5-q95 quantile range of 125 to 480, while the ranges for the CAMI-gut, CAMI-skin, and CAMI-oral datasets are 202-281, 162-282, and 162-281, respectively (Table S7). This high variability in insert size distributions among real datasets is likely caused by differences in Illumina library preparation methods and possibly other technical factors. Importantly, these findings suggest that the CAMI datasets lack some realism when considering the full diversity of metagenome datasets. Our metagenome simulation pipeline was based on the established CAMI simulation methodology, so our training dataset also lacked some realism (q5-q95 range of 178-372), which likely reduced the ability of our model to generalize across the diversity of real metagenome datasets.

In order to improve ResMiCo generalization to metagenomes with highly varying insert size distributions, we augmented the training dataset with simulations

incorporating larger insert sizes (mean=450, sd=120) and smaller insert sizes (mean=190, sd=75) distribution. These added simulations doubled the size of the dataset from 40M to 80M contigs. We retrained ResMiCo on a random subset of 3000 metagenomes containing 52M contigs (66% of all generated samples) in order to fit the data into the 1.3T space available on the compute nodes on our HPC cluster. The model was trained for 50 epochs, and the best performance on the validation set (AUPRC 0.715) was achieved at the 46th epoch. The newly added simulation data is publicly available at http://ftp.tue.mpg.de/ebio/projects/ResMiCo/.

We found that our retrained ResMiCo model better generalizes to metagenomes with more variable insert size distributions than observed in the human-associated CAMI datasets (Tables S5 & S6). All figures and tables in the manuscript have been regenerated with our new model, and the ResMiCo python package now includes the updated model (https://github.com/leylabmpi/ResMiCo). Moreover, given that the new model can better generalize to more variable insert size distributions, we have expanded the inclusion criteria for evaluating real metagenomes (see "Data preprocessing" in the Materials and Methods) and thus included more real metagenomes in our estimation of misassembly rates among metagenome datasets (see "ResMiCo detects a 3-12% misassembly rate in real-world metagenomes" in the Results and also Table S8).

5) More analysis of the correlation of true and ResMiCo's error rates with different assembly parameters should be done to check for systematic biases. From what is there now, the statement on N50 as an assembly quality measure in Fig 5b needs more elaboration as there is 0.9 correlation between true error rate and N50 where samples are actually well assembled (richness=50, seq_depth=8000000) which can also be seen from a completely non-overlapping y-axis for N50 (1-3k vs 5-25k). Also, is it correct to assume from Fig 5a that ResMiCo detects approximately twice the true error rate? The effect of this needs to be addressed and explained.

We refer to N50 as a contiguity measure, which is typically the main optimization criterion for benchmarking metagenome assemblers on real data, given the lack of ground truth for such datasets. This practice of optimizing for contiguity can lead to the impression that assembly contiguity is always highly correlated with assembly quality. However, we show that highly contiguous metagenomes can still have relatively high misassembly rates. For example, the most well assembled metagenomes (low richness and high sequencing depth) show a strong correlation (0.9) between N50 and the true error rate (many misassemblies), see Fig 5b. In other instances, we found contiguity to not strongly correlate with the true error rate. These findings demonstrate that optimizing for assembly contiguity does not necessarily also optimize for accuracy. Given that we show how ResMiCo performance is robust to assembly parameters (e.g., kmer lengths), ResMiCo enables robust optimization for assembly accuracy on real metagenomes that lack a ground truth (see "Assembly optimization based on ResMiCo-identified error rates" in the Results).

Specifically in regards to the reviewer's question: "is it correct to assume from Fig 5a that ResMiCo detects approximately twice the true error rate", we found that for most of the simulation parameters, the ratio between the number of misassemblies identified

by ResMiCo and the true amount is 1.1. We note that this is a result of selecting a threshold at 0.8, which corresponds to a recall of 0.72 and a precision of 0.65. However, the ratio depends on richness, sequencing depth, abundance distribution and read length (Fig S10). We do not consider this problematic, given that we consider the case where the data (corresponding to one set of the conditions) is already collected and assemblers are applied on the same set of reads. While the absolute number can be overestimated, the ranking based on the true error rate (not available to the user) and based on the ResMiCo scores are highly correlated.

Both of the points described above are now clarified in the manuscript in the "Assembly optimization based on ResMiCo-identified error rates" section of the Results.

6) Specification of compute and memory requirements and comparison to other tools is missing in the manuscript. Especially, given the note in the GitHub page about the strong preference for GPU rather than CPU for running ResMiCo it is necessary to specify how much of compute resource it takes to run per metagenome assembly.

We benchmarked the ResMiCo model with direct comparisons between utilizing one CPU versus one GPU. On the CAMI2-gut dataset (10 metagenomes), ResMiCo was >2X faster with a GPU versus a CPU (108 +/- 0.7 versus 38.7 +/- 10.3 contigs per second). While a GPU is substantially faster than a CPU, one could still process nearly 140k contigs in 1 hour with a single CPU. Nonetheless, we note that multiple GPUs are recommended for training the model on large datasets, given that training on CPUs would not be feasible with the size of the dataset used in this work.

We have updated the Materials and Methods and the README in the GitHub repository to include this information.

7) L221 "The prediction for the contig was obtained by selecting the maximum score across all chunks" - The true probability of having a misassembly in a contig is not equal to a maximum probability of one of 20 kbp chunks of a contig. Therefore, it should be a product of probabilities that there is no misassembly in the chunk (1-x). For example, if there are two chunks with 60% and 40% probability of misassembly then the combined probability should be,

1 - (1 - 0.6) * (1 - 0.4) = 0.76, 76%

Another issue is adjusting this to strides between chunks. In addition, it is also not explained why there should be a stride between chunks of long contigs.

When this change is introduced the percentage of misassembled contigs will probably increase.

The goal of our work is not to predict misassembly probability (a continuous value), but to optimize model accuracy to detect misassemblies (a binary value). These 2 criteria do not necessarily fully align. In this work, we selected a threshold of 0.8 to mark misassemblies, but the observed misassembly rate is lower than 80% (precision = 0.65).

With this said, we found the reviewer's idea of aggregating scores across chunks to be compelling and investigated this strategy.

In order to implement the reviewer's proposed method, we had to split the contigs into chunks with 0 overlap, which differs from our existing method of using an overlap of 500 bp between contig chunks for long contigs in order to mitigate problems when the breakpoint is located at the end of a chunk (edge effects), given that the two consecutive chunks may individually look like correctly assembled contigs. However, we rarely observed this theoretical scenario in the data, which is reflected by a similar AUPRC on the validation set regardless of whether the 500 bp overlap was implemented (AUPRC with overlap: 0.72724; AUPRC without: 0.72721). Therefore, we considered this change to the methodology insubstantial to the goals of this work and further evaluated the reviewer's proposed method.

When we directly applied the formula proposed by the reviewer to the ResMiCo output, we obtained an AUPRC of 0.723, which is lower than 0.727 achieved with our method of computing the maximum score of all contig chunks.

Given that the output of a neural network (NN) is not calibrated by default (Chuan Guo, 2017 arXiv:1706.04599v2), we calibrated the ResMiCo model with isotonic regression in order to transform the score to probability. This process involved splitting the validation set randomly into two parts: one to fit isotonic regression and another to compare performance of the two methods. The AUPRC of this new method was slightly lower than our existing method (0.721 vs 0.726), suggesting that the reviewer's proposed method does not result in improved classification accuracy. While the reviewer's proposed method may be quite useful to detecting specific (multiple) misassembly locations within contigs, robustly assessing this possible application is out of the scope of this work. Therefore, we have retained our original method for detecting misassemblies in the paper.

8) In the manuscript, the authors use the absolute counts of misassembled contigs for statistical purposes. However, it is possibly more accurate to normalise the misassembly rate per length (kbp) of contigs unless authors can provide reasons for why looking only at the absolute count is sufficient.

We computed "misassemblies length", which we define as the sum of misassembled contig lengths divided by total number of bases. We have added this statistic to Table 2, where we describe the datasets used in the paper and to Table 3, where we calculate the statistic before and after filtering out contigs identified as misassemblies by ResMiCo. We also study how the misassembly rate and model performance are distributed across contig lengths in Fig S4.

9) I could not install ResMiCo and below are the outputs of commands as described in the github repo.

Attempt to install via conda:

```
$ conda create -n resmico_env bioconda::resmico
Collecting package metadata (current_repodata.json): done
Solving environment: failed with repodata from
current_repodata.json, will retry with next repodata
source.
Collecting package metadata (repodata.json): done
Solving environment: -
```

Attempt 2 to install via conda:

```
$ conda create -n resmico_env resmico
Collecting package metadata (current_repodata.json): done
```

```
Solving environment: failed with repodata from
    current_repodata.json, will retry with next repodata
    source.
    Collecting package metadata (repodata.json): done
```

Attempt to install via pip:

```
$ pip install resmico
 Collecting resmico
 ERROR: Could not find a version that satisfies the
 requirement resmico (from versions: none)
 ERROR: No matching distribution found for resmico
```

We apologize for the challenges that the reviewer encountered when trying to install and utilize ResMiCo. Prior to the original submission of this manuscript, we made a strong effort to develop a robust and easy-to-use python package, with continuous integration testing and availability via both pypi and bioconda. Still, the C++ code utilized to greatly improve the computational efficiency of converting BAM files to features for ResMiCo has led to installation issues for some users. We have updated the ResMiCo package to reduce such issues, and we will continue to make improvements as needed. We encourage all users to submit issues at the GitHub repository, and we try to respond rapidly to such issues.

Other minor comments [with square brackets for suggested edits]:

1) Note in GitHub could be more clear: “Note: Although ResMiCo can be run on a CPU, the performance is orders of magnitude wors[e than on a GPU], so we only recommend running on CPU for testing.”

We have updated the README accordingly.

2) FigS1 - add a note that these are “true” misassemblies predicted by MetaQUAST.

We have updated the figure caption accordingly.

3) L55 “collection [of] gut metagenomes”

We have fixed the typo.

4) L76 Please add the explanation that the “insert size” is the distance between two paired reads as this might not be well known to everyone and might lead to misunderstanding that it is the size of insertion that is a misassembly.

We have clarified this point in this location in the text.

5) Mistake on L269 - “the evaluation [of] metagenomics analysis tools.”

We have corrected the text.

6) L93 “The pool was also filtered by CheckM-estimated completeness (≥ 90%) and contamination (≤ 5%). Other filtering criteria included: i) only ”high” MIMAG quality” - It is redundant to mention “CheckM-estimated completeness (≥ 90%) and contamination (≤ 5%)” filters together with “high” MIMAG quality standard as these CheckM filters are included in “high” MIMAG standard.

Not all readers are aware of the criteria included in the MIMAG quality standard, so we argue that an explicit description of the assembly filtering criteria used in our methods is more suitable than implicit descriptions which require more background knowledge by the reader.

Reviewer 2

This paper addresses the major challenge of evaluating contigs misassembly from the metagenomic reads without reference requirements. The proposed system, ResMico, utilizes a novel deep convolutional neural network with skip connections between non-adjacent layers. The authors have performed extensive evaluations through simulated datasets representing variously plausible microbial communities and sequencing parameters utilizing reference bacterial and archaeal genomes from the Genome Taxonomy Database. They showed the superior performance (accuracy and robustness) of ResMico compared to the existing methods, including DeepMAsDB, the tool previously developed by the authors’ group. They further

showcased ResMiCo's ability to detect an average of 4.7% error rate of contigs/metagenome in a large collection of gut metagenomes. The paper presents a tool with significant utility in metagenomics research. It is generally well-written; however, some technical aspects could be better explained.

We thank the reviewer for their constructive comments.

1) The Residual block structure was used in ResMiCo. However, more detailed descriptions are needed to clarify the rationale for using it, and how the number of RGs was determined. Please also indicate in the Fig.2 caption that 14 represents the number of the selected features in the input to the neural network.

We wrote that 14 represents the number of the selected features in the input to the neural network in the caption of Fig 2.

Residual connections enable deeper NNs (i.e., more layers) and more efficient training compared to classical convolutional NNs. Moreover, deeper models can capture more complex patterns spread over longer inputs. The number of residual blocks within a group, number of groups, and other model design choices were tested by training on the subset of the n9k-train dataset and then comparing the performance on the validation set. The list of optimized hyperparameters and the attempted values are provided in Table S2.

We describe the process of selecting the best performing model in a supplemental section entitled "NN architecture selection". First, we selected the best performing neural network architecture type. For this, we constructed a deep convolutional NN, a bidirectional recurrent NN with LSTM units and with GRU units, a transformer encoder, and a residual convolutional NN. All models had roughly 0.5 million parameters. The residual convolutional NN outperformed the other network architectures for all contig lengths and was thus selected for this work. All hyperparameter optimizations were applied to this architecture only.

2) P9: Clarify why MetaSPAdes (but not MEGAHIT) was used for assembling the published, real-world metagenomes.

We solely utilized metaSPAdes for real-world datasets, given that i) evaluation of both assemblers adds substantial computation and complexity to the evaluations and ii) metaSPAdes is generally more accurate than MEGAHIT, so metaSPAdes should be preferred over MEGAHIT. We have clarified this point in the Materials and Methods section, in the location referenced by the reviewer.

3) P12: the number of contigs used in the feature importance evaluation seems relatively small: 200 contigs randomly selected from the correct and incorrect. There is a concern if the size is sufficiently large for a good assessment of the importance. Could you discuss further on this?

Given the high computation burden of SHAP, we assessed the usefulness of applying the method to a larger number of contigs by progressively applying SHAP to less contigs, with the rational that if the number of contigs substantially affected SHAP results, we should see a substantial change in the results as the number of contigs declined. In contrast, the overall rankings were stable when applying SHAP to 200, 100, 50, 20, or 10 contigs. We note that the absolute importance values did vary across these varying runs, but the overall rankings suggest that SHAP was consistent at the broad scale. Notably, the runs with 10 and 20 contigs disagree only in one feature which to discard. Error bars in Fig 3 and Fig S12 correspond to the stdev computed over 5 runs of selecting contigs.

We agree with the reviewer that sample size can affect model interpretability in some cases, including in this work. Therefore, we have added Fig S12 with our findings from this analysis in order to show that altering the number of contigs does not change the selection of important features.

4) P15: Using UMAP to examine how data were represented by ResMiCo is a good idea. However, as we know, UMAP only allows a visual inspection. I wonder if clustering the misassembled contigs would generate additional insights on the type of misassemblies in the data and if ResMiCo makes more mistakes in classification for any kind of misassembles represented by some cluster.

To test the reviewer's idea, we clustered contigs based on their representations in the ResMiCo model and assessed whether these clusters matched the grouping of contigs by misassembly type. A strong overlap of these 2 clustering methods would suggest that the misassembly type could be classified simply by using the clustering of model representations.

We note that there are 4 types of misassemblies reported by MetaQUAST: interspecies translocation, relocation, inversion, and translocation. Inversions were only 0.1% of all misassemblies, so we did not include them in the following analysis.

To represent contigs as vectors of the same size, we produced embeddings from the ResMiCo pooling layer (the same as for UMAP). We randomly sampled 1 million contigs from each dataset and used only misassemblies for the following analysis. We used k-means clustering to generate 3 clusters for the n9k-train dataset and predicted cluster assignments for the n9k-novel test dataset. To compare the two clusterings (based on misassembly type and on k-means from the embeddings), we computed Clustering Purity (0.52) and Adjusted Rand Index (0). These finds demonstrate that the model embeddings do not strongly correspond with misassembly types and so one can not use clustering of the embeddings to determine misassembly type for real-world data.

To get some insights into contig characteristics that are the most challenging for ResMiCo, we also fitted k-means with 50 clusters. Clusters with less than 100 contigs were filtered out for clarity of the results, so the resulting number of clusters was 35 clusters. We describe contigs with features used to simulate the data (i.e., sequencing depth, community richness, assembler type, and read length), with misassembly types reported by MetaQUAST (i.e., interspecies translocation, relocation, and translocation)

and the most informative (according to SHAP) positional features (i.e., mapping quality, mean alignment score, mean insert size, aand coverage), and also contig length. The Table S9 shows mean values of each characteristic for each cluster and is sorted based on average ResMiCo score. At the bottom of the table, there are clusters of misassemblies that are challenging to accurately detect with ResMiCo. These clusters have the following characteristics: high median contig length, more translocation misassembly types, and low richness. The same can be concluded from the other figures (Fig S4 & S5 for contig length, Fig S7 for misassembly type, and Fig S9 for richness) and is discussed in the Results section. However, exceptions could be found, such as Cluster 29, which had an average ResMiCo score of 0.53 but from the high level characteristics, the cluster looked quite similar to the clusters with substantially high average scores.

Reviewer 3

In this paper, the authors present ResMiCo, a deep convolutional neural network that is trained to identify misassembled contigs in a reference-free manner, from metagenome assemblies. While metagenome assemblies are rapidly accumulating, the quality of the assemblies can still be much improved in many cases. In this respect, it is critical to identify and correct misassembled contigs from existing metagenomes. As per results, ResMiCo outperforms prior methods *greatly*. While this is very positive, we have remained not yet fully convinced in terms of the experiments that support the idea of superiority. In the following some major comments.

* In lines 285-286, "This filtering resulted in a reduction of the true error rate from 4% to 1% while keeping the contiguity metrics virtually unmodified." How exactly do you estimate the error rate?

The misassembly labels produced by MetaQUAST for all contigs in the n9k-novel dataset, so we can measure the true error rate before and after filtering. We have clarified this point in the text.

* In Metaquast, how does Genome fraction change after filtering out misassembled contigs?

We evaluated this question with the CAMI-gut and CAMI-marine datasets, given that known reference genomes must be used in order to obtain an accurate measure of genome fraction. The genome fraction did not substantially change for either dataset when comparing post- versus pre-contig filtering by ResMiCo (Wilcox, P ≥ 0.66 for both datasets). We have added these data as Figure S11 and updated the Results to include this information.

* It would be clearly favorable to evaluate the method on real data that have a standard reference. Suggestions are: the Bmock12 data set in Volkan Sevim's work treating shotgun

metagenome data reflecting a mock community, sequenced using Oxford Nanopore, PacBio and Illumina technologies. Another dataset is the NWCs (natural whey culture) data set in Vincent Somerville's "Long-read based de novo assembly of low-complexity metagenome samples results in finished genomes and reveals insights into strain diversity and an active phage system." In both works, short reads and standard references are provided, where the latter was generated using hybrid assembly methods.

While mock communities provide real-world sequence data, the community richness of such communities described in the literature (generally ~20 strains) is orders of magnitude lower than gut, soil, or other complex, natural microbial communities (1000s of species/strains; https://doi.org/10.1111/rssc.12206, https://doi.org/10.1038/ismej.2013.10). Given that richness strongly affects assembly accuracy (simple communities generally assemble with higher completion and accuracy), we argue that the evaluation of mock communities does not provide meaningful insight into the real-world performance of ResMiCo.

We provide a summary of many of the published mock communities, in order to give a sense of the richness (number of strains) in such communities:

- **12 strains (BMock12): https://doi.org/10.1038/s41597-019-0287-z**
- **20 strains: https://doi.org/10.1128/spectrum.01915-21**
- **20 strains: https://doi.org/10.1186/s40168-021-01048-3**
- **19 strains: https://doi.org/10.1038/nbt.3601**
- **21 strains: https://doi.org/10.1186/s12866-015-0351-6**
- **23 strains: https://doi.org/10.1038/sdata.2016.81**
- **26 strains: https://doi.org/10.1038%2Fsdata.2016.81**
- **8 strains: https://doi.org/10.3389/fmicb.2020.00953**
- **10 strains: https://doi.org/10.1038/nbt.3960**

* Please provide Genome Fraction as an evaluation category generated by Metaquast. Since metagenomes often contain various strains referring to identical species, it is important to have a look at how Genome Fraction is affected. For example, losing contigs from particular strains -- indicated by a drop in Genome Fraction -- would imply undesired biases.

As described above, we evaluated the change in MetaQUAST-calculated genome fraction with 2 datasets: CAMI-gut and CAMI-marine. The genome fraction did not significantly change for either dataset (Wilcox, $P \geq 0.66$ for both datasets). These findings are now conveyed in Figure S11 and in the Results section.
