# Supplementary material for: ResMiCo: Increasing the quality of metagenome-assembled genomes with deep learning
Source: PLoS Comput Biol. 2023 May 1;19(5):e1011001. doi: 10.1371/journal.pcbi.1011001 (PMC10174551; doi:10.1371/journal.pcbi.1011001)
Supplement: Attachment — Submitted filename: response_to_reviewers.pdf [file pcbi.1011001.s006.pdf]

Reviewer #1

Thanks to the authors for addressing all of my concerns and comments from the first review in the revised manuscript. I have no remaining questions and wish the tool will be well maintained and developed continuously in the future.

We thank the reviewer for their helpful comments, which have substantially improved the quality of this work.

Reviewer #2

All my comments and suggestions have been satisfactorily addressed.

We thank the reviewer for their helpful comments, which have substantially improved the quality of this work.

Reviewer #3:

I disagree with the authors on not running the method on mock communities. While these mock communities are not *real-world* data, they are *real* data, and provide -- as widely accepted by the community -- a basic test that a method does not perform in an erratic manner when being confronted with real data. (thanks for listing the communities, but I am aware of everything you mention in your response anyway)

So, please run your method on mock communities, or convince me otherwise that it does not behave in mistaken ways when confronted with *real* data. (there was no mentioning in the response that you had done this; and I sometimes had the feeling that there was some confusion about the difference between *real-world* and *real* data).

As we described in our initial response, existing mock communities contain orders of magnitude less richness than complex communities from biomes such as soil or the mammalian gut. Moreover, the inter-genome similarity is not reflective of most real-world communities, given that mock communities lack intra-species diversity (i.e., only one genome per species in mock communities). These factors generally result in metagenome assemblies of unrealistically high quality.

Despite these substantial caveats, we agree with the reviewer that mock communities provide the best real-world datasets in which the ground truth is fully known. Therefore, we evaluated 2 mock communities: BMock12 and MBARC-26. Despite the low number of truly misassembled contigs, ResMiCo performance was in line with our expectations based on simulations of low richness communities (Figures S8 & S9), with an AUPRC of 0.475 and an AUROC of 0.951 when averaged across the 2 datasets (Table S9). We have added a description of these new findings to the Results subsection: "ResMiCo outperforms existing models and is robust to metagenome novelty" and added

a new supplemental table that summarizes these new results (Table S9). The Methods have also been updated with a description of the mock community dataset retrieval.
